# Fruquintinib as first‐line or second‐line treatment in unresectable or metastatic soft‐tissue sarcoma: A prospective, single‐arm phase II study

**DOI:** 10.1002/ctm2.70308

**Published:** 2025-04-15

**Authors:** Xiaowei Zhang, Ting Zhao, Biqiang Zheng, Wangjun Yan, Yong Chen, Yu Xu, Chunmeng Wang, Junhua Zhang, Jian Wang, Lin Yu, Xin Liu, Zhiguo Luo

**Affiliations:** ^1^ Department of Medical Oncology Fudan University Shanghai Cancer Center Shanghai China; ^2^ Department of Oncology Shanghai Medical College Fudan University Shanghai China; ^3^ Department of Musculoskeletal Surgery Fudan University Shanghai Cancer Center Shanghai China; ^4^ Department of Radiation Oncology Fudan University Shanghai Cancer Center Shanghai China; ^5^ Department of Pathology Fudan University Shanghai Cancer Center Shanghai China

1

Dear Editor,

We conducted the first prospective, single‐arm phase II study and confirmed that fruquintinib is an effective and well‐tolerated chemo‐free regimen for the first‐line or second‐line therapy of patients with unresectable or metastatic soft‐tissue sarcoma (STS), particularly for those with the histopathologic subtype of angiosarcoma (AS).

STS are rare tumours that comprise over 50 distinct histologic subtypes, accounting for 1% of all adult malignancies.^1^, ^2^ For individuals with unresectable or metastatic STS, the median overall survival (OS) varies between 12 and 16 months, while the 5‐year OS rate is 60%–80%.^3^ Over the past decade, anti‐angiogenic therapy has been utilised to treat advanced STS.^4^ Pazopanib was the first tyrosine kinase inhibitor approved for advanced STS,^5^ followed by regorafenib and anlotinib, both of which have shown excellent performance in the treatment of advanced STS.^6^

Fruquintinib, a multi‐target inhibitor of VEGF receptors 1, 2 and 3, has shown overall response rate (ORR) of 13.1% and 17.7% in two retrospective studies^7^ underscoring its promise as a chemo‐free treatment for advanced STS. However, due to the inherent limitations of retrospective studies, further investigation is necessary to assess the efficacy and safety of fruquintinib in unresectable or metastatic STS.

In our study, a total of 31 individuals were evaluated for eligibility and assigned to receive fruquintinib monotherapy (Figure 1A). Details of the study design and statistical analysis are described in Supporting Information. All patients were aged between 19 and 78 years. Among them, there were 15 (48.4%) males and 16 (51.6%) females. In the total population, 22 (71.0%) participants had distant metastases at first diagnosis, while another nine (29.0%) were categorised as unresectable STS without metastases. Among the 22 metastatic STSs, 15 (68.2%) had metastatic lesions in two or more organs. The most common primary lesions were trunk and extremity (36.7%), followed by lung (20.0%), brain (16.7%), head (13.3%), spleen (10.0%) and breast (3.3%). During metastasis, most cases spread to lungs (63.6%), followed by other sites including bones (50.0%), lymph nodes (36.4%), liver (27.3%), pleura (13.6%) and muscle (9.1%). Of them, 18 (58.1%) underwent prior surgery, 12 (38.7%) received prior first‐line therapy and four (12.9%) received prior radiotherapy (Table S1).

**FIGURE 1:**
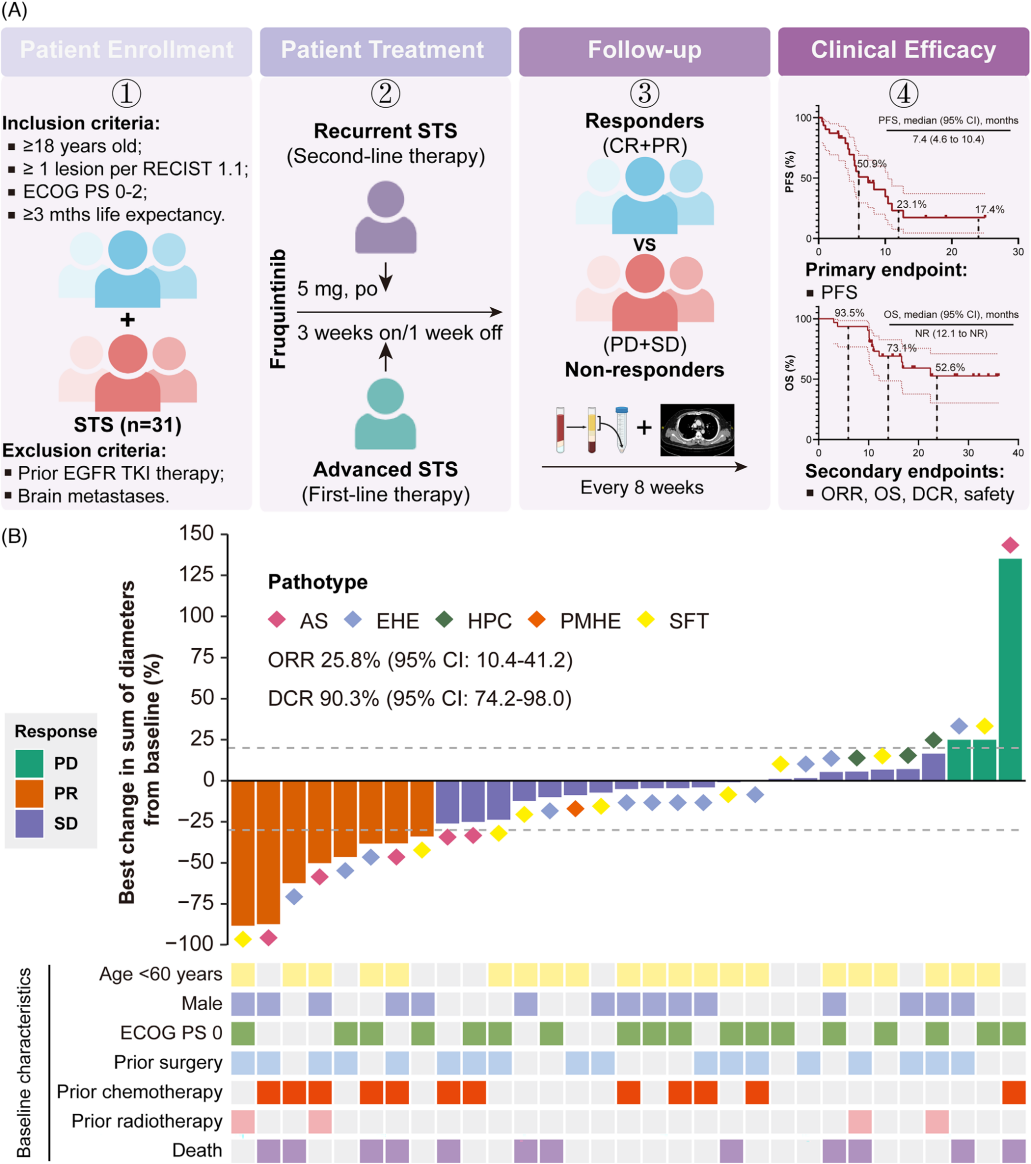
Work flow and maximum percent change in sums of diameters from baseline in target lesions. (A) This study (ClinicalTrials.gov, NCT05142631) was designed as a prospective, single‐arm phase II study evaluating the efficacy and safety of fruquintinib as first‐line or second‐line treatment in patients with unresectable or metastatic soft‐tissue sarcoma (STS). Between 4 November 2021 and 25 April 2024, a total of 31 patients were enrolled. The primary endpoint was progression‐free survival (PFS), with the secondary endpoints being the objective response rate (ORR), disease control rate (DCR) and overall survival (OS), as well as safety. (B) Waterfall plot of best change in the sum of diameters in target lesions from baseline according to RECIST 1.1. AS, angiosarcoma; EHE, epithelioid haemangioendothelioma; HPC, haemangiopericytoma; PD, progression disease; PMHE, pseudomyogenic haemangioendothelioma; PR, partial response; SD, stable disease; SFT, solitary fibrous tumour.

As of the data cutoff on 26 November 2024, patients received between 1 and 35 treatment cycles, with a median of eight cycles. The median follow‐up duration was 19.2 months (95% confidence interval [CI]: 16.3‒34.0). Among them, 18 patients (58.1%) experienced progression‐free survival (PFS) events, and 12 patients (38.7%) died. ORR was 25.8% (95% CI: 10.4‒41.2), and the disease control rate (DCR) was 90.3% (95% CI: 74.2‒98.0) (Figure 1B). Median PFS was 7.4 months (95% CI: 4.6‒10.4), with the 12‐ and 24‐month PFS rates were 23.1% and 17.4%, respectively (Figure 2A). Median OS was not reached (NR) (95% CI: 12.1 to NR); the 12‐ and 24‐month OS rates were 73.1% and 52.6%, respectively (Figure 2B). Among the five different histologic subtypes of STS, patients with AS exhibited the highest ORR of 50% (95% CI: 11.8‒88.2) (Figure 2C). However, the wide 95% CIs reflect statistical uncertainty from small sample sizes in certain subtypes, necessitating further validation. In subgroup analysis, patients with low level of baseline systemic immune inflammation index (SII) exhibited better PFS (*p* = .0105) and more favourable OS (*p* = .1634) compared to those with high level of SII (Figure 2D,E). SII may serve as predictors to monitor tumour progression in STS treatment. Unfortunately, no differences were observed in PFS and OS among patients stratified by the median of systemic inflammation response index (Figure 2F,G).

**FIGURE 2:**
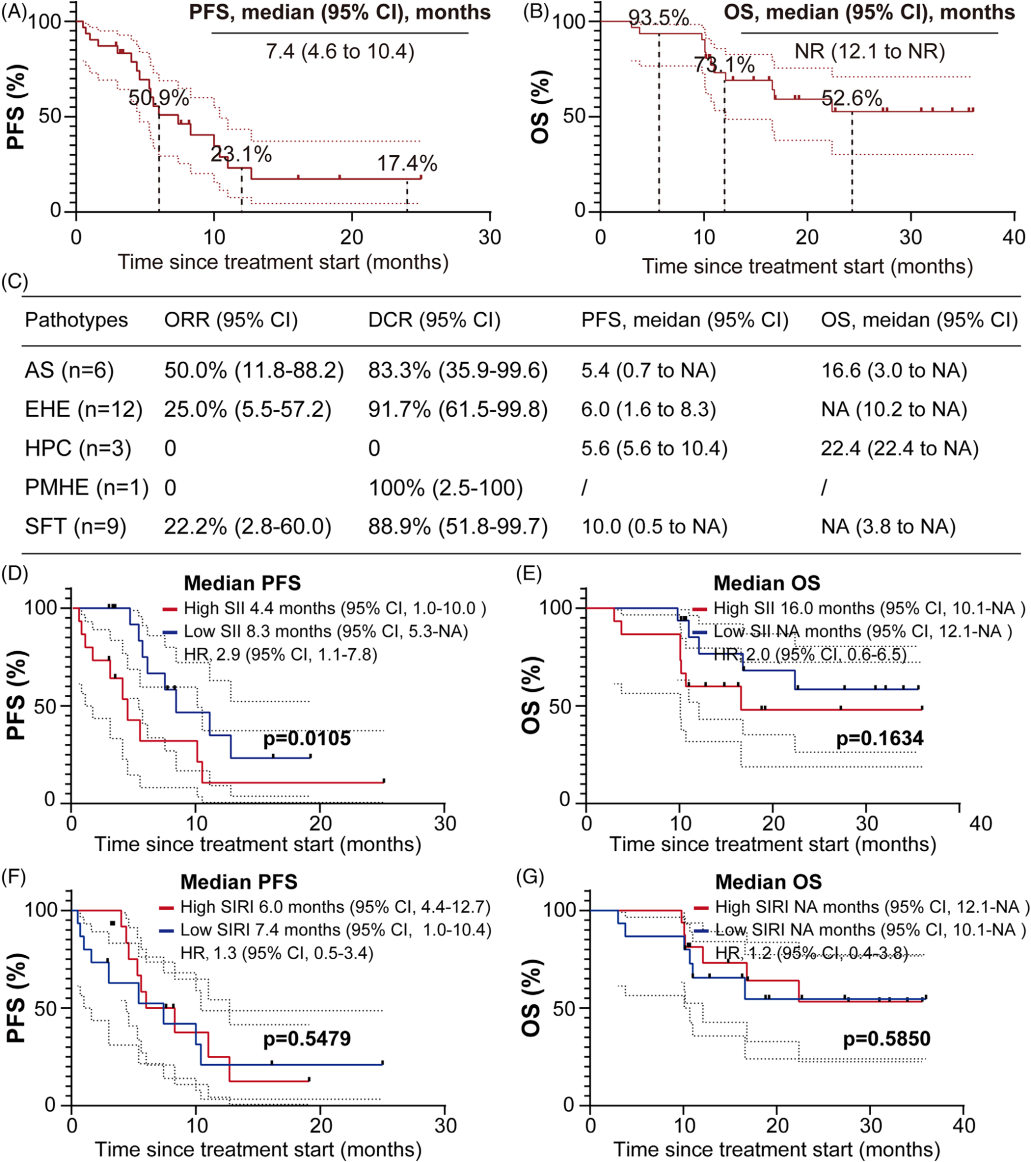
Kaplan‒Meier curves of progression‐free survival (PFS) (A) and overall survival (OS) (B) in the overall study population and the five different histologic subtypes (C), as well as stratified by systemic immune inflammation index (SII) (D and E) and systemic inflammation response index (SIRI) (F and G). AS, angiosarcoma; DCR, disease control rate; EHE, epithelioid haemangioendothelioma; HPC, haemangiopericytoma; NR, not reached; ORR, objective response rate; PD, progression disease; PR, partial response; PMHE, pseudomyogenic haemangioendothelioma; SFT, solitary fibrous tumour.

In the safety analysis, 29 (93.5%) participants had at least one treatment‐related adverse events (TRAEs) (Table 1). The most commonly observed TRAEs of any grade, regardless of causality, were hypertension (54.8%), hand‒foot skin reaction (29.0%), fatigue (25.8%), epistaxis (25.8%) and oral mucositis (25.8%). Grade 1–2 toxicities occurred in 29 individuals (93.5%) and included hypertension (35.5%), palmar‒plantar erythrodysesthesia syndrome (25.8%), fatigue (25.8%), epistaxis (25.8%) and oral mucositis (25.8%). Grade 3–4 toxicities were observed in eight (25.8%) cases, including hypertension (19.4%), liver dysfunction (3.2%) and palmar‒plantar erythrodysesthesia syndrome (3.2%). Hypertension was the most common TRAE, aligning with findings from previous studies.^8^ Treatment interruptions were observed in three patients (9.7%) due to grade 3–4 hypertension, while 14 patients (45.2%) experienced a dose reduction of 1 mg as a result of TRAEs. The reasons for dose reduction included grade 3–4 hypertension (*n* = 6), hand‒foot syndrome (*n* = 5), grade 3–4 liver injury (*n* = 2) and grade 2 proteinuria (*n* = 1). No treatment‐related deaths were observed.

**TABLE 1:** Summary of adverse events for eligible patients with unresectable or metastatic soft‐tissue sarcoma (STS).

Category	All grades	Grade 1‒2	Grade 3‒4
ALT level elevated	1 (3.2%)	0	1 (3.2%)
AST level elevated	1 (3.2%)	0	1 (3.2%)
Decreased appetite	4 (12.9%)	4 (12.9%)	0
Diarrhoea	2 (6.5%)	2 (6.5%)	0
Dysphonia	3 (9.7%)	3 (9.7%)	0
Fatigue	8 (25.8%)	8 (25.8%)	0
Hand‒foot syndrome	9 (29.0%)	8 (25.8%)	1 (3.2%)
Hypertension	17 (54.8%)	11 (35.5%)	6 (19.4%)
Hypothyroidism	2 (6.5%)	2 (6.5%)	0
Proteinuria	1 (3.2%)	1 (3.2%)	0
TSH level elevated	4 (12.9%)	4 (12.9%)	0
Back pain	4 (12.9%)	4 (12.9%)	0
Epistaxis	8 (25.8%)	8 (25.8%)	0
Joint pain	4 (12.9%)	4 (12.9%)	0
Oral mucositis	8 (25.8%)	8 (25.8%)	0
Rash	4 (12.9%)	4 (12.9%)	0
Limb pain	6 (19.4%)	6 (19.4%)	0
Gingivitis	5 (16.1%)	5 (16.1%)	0

Abbreviations: ALT, alanine aminotransferase; AST, aspartate aminotransferase; TSH, thyroid‐stimulating hormone.

To identify key independent factors associated with PFS and OS, we utilised Cox proportional hazards regression analysis. Multivariate Cox analysis revealed that aged under 60 years, no prior surgery and a higher Eastern Cooperative Oncology Group (ECOG) performance status (PS) score were associated with worse PFS (Figure 3A). Meanwhile, female gender, non‐AS histologic subtype, absence of prior surgery, no prior radiotherapy and a higher ECOG PS score were risk factors associated with unfavourable OS (Figure 3B).

**FIGURE 3:**
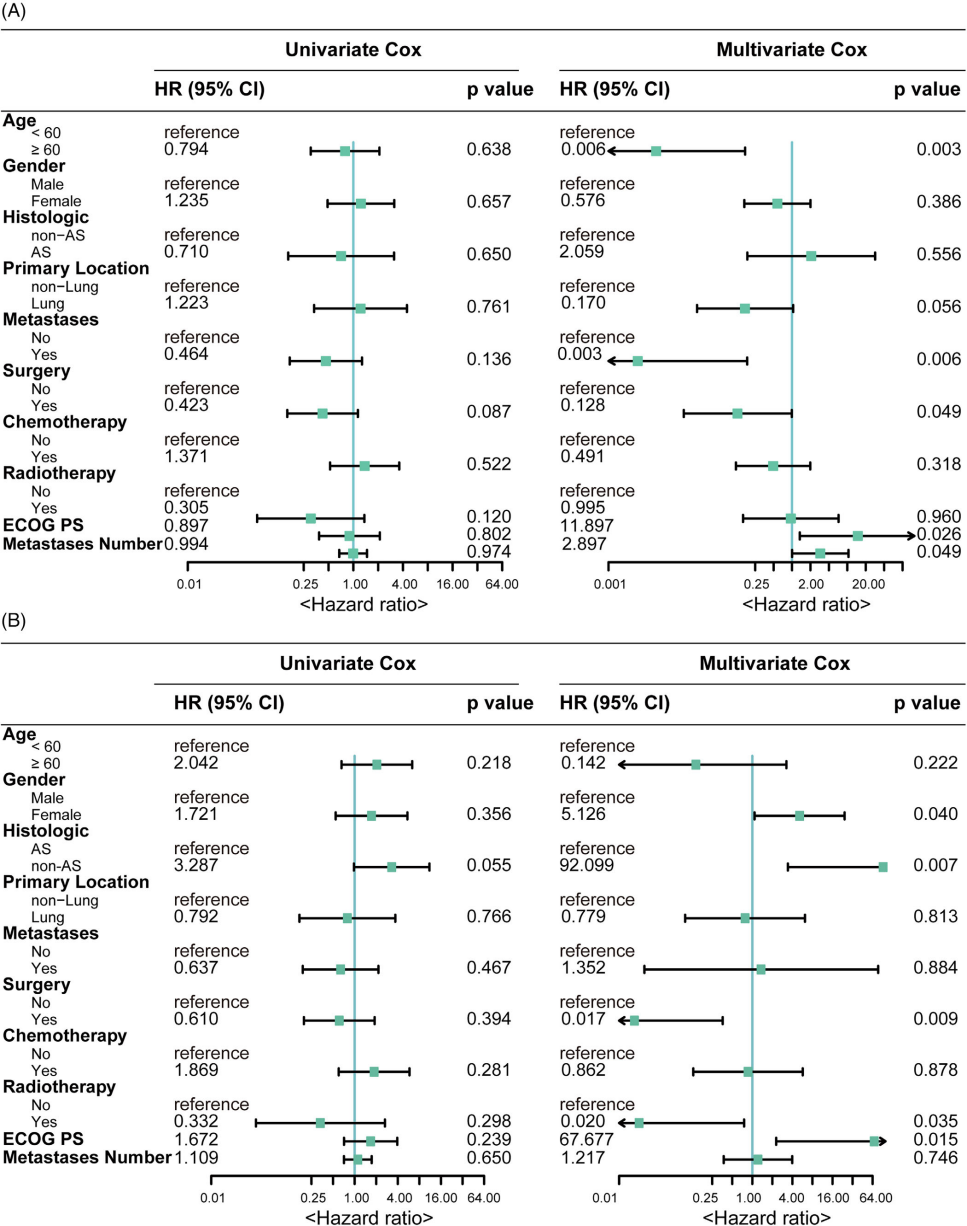
The univariate and multivariate Cox regression analysis of baseline characteristics associated with progression‐free survival (PFS) (A) and overall survival (OS) (B) in the study population. AS, angiosarcoma; ECOG PS, Eastern Cooperative Oncology Group performance status.

In summary, the efficacy observed in our study was superior to that reported in two previously published retrospective studies.^9^, ^10^ Our study demonstrated that first‐line or second‐line fruquintinib therapy was an effective regimen with a predictable safety profile for unresectable or metastatic STS.

However, this clinical trial still has limitations. First, this is a single centre, single‐arm clinical trial with 31 patients. Findings of this study require validation in larger trials or in multicentre. Second, we only included five histologic subtypes of STS, with limited numbers of haemangiopericytoma and pseudomyogenic haemangioendothelioma. Therefore, results of this study lack of good generalisability. Finally, we only identified baseline SII as a biomarker related to efficacy, which provides insufficient references for prognosis evaluation, and follow‐up for unresectable or metastatic STS.

In conclusion, despite the limitations of a single‐arm design, this study suggests that fruquintinib is an effective and manageable chemo‐free treatment for unresectable or metastatic STS, especially for the AS histologic subtype. Additionally, baseline SII serves as an effective tumour marker in the surveillance of STS progression.

## AUTHOR CONTRIBUTIONS

Xiaowei Zhang, Ting Zhao, Xin Liu and Zhiguo Luo contributed to the conception and design, methodology, data collection and analysis, as well as manuscript writing and final approval. Biqiang Zheng, Yu Xu, Yong Chen, Chunmeng Wang, Wangjun Yan, Jian Wang and Lin Yu were responsible for patient provision, data curation, investigation and methodology. All authors participated in the manuscript preparation and approved the final version.

## CONFLICT OF INTEREST STATEMENT

The authors declare they have no conflicts of interest.

## ETHICS STATEMENT

This study was approved by the Ethics Committee of Fudan University Shanghai Cancer Center. Participants provided written informed consent prior to participation.

## Supporting information



Supporting Information

Supporting Information

## Data Availability

The data supporting the findings of this study are available from the corresponding author upon reasonable request.
