# Supplementary material for: Fruquintinib as first‐line or second‐line treatment in unresectable or metastatic soft‐tissue sarcoma: A prospective, single‐arm phase II study
Source: Clin Transl Med. 2025 Apr 15;15(4):e70308. doi: 10.1002/ctm2.70308 (PMC12000221; doi:10.1002/ctm2.70308)
Supplement: Supplementary file 1 — Supporting Information [file CTM2-15-e70308-s001.docx]

**PATIENTS AND METHODS**

**Study Design and Patients**

In this prospective phase II study, we applied a Simon’s optimal two-stage design to assess PFS as the primary endpoint. To achieve the hypothesized PFS of 4.9 months with fruquintinib treatment, a total of 28 patients were required to ensure 80% power at a two-sided α level of 5%. Considering a dropout rate of 10%, we set target sample size at 31 patients. Sample size calculation was performed using the EAST software.

Eligible patients were ≥18 years old, weighed at least 40 kg, and were required to provide informed written consent before participating in the clinical trial. All patients had unresectable or metastatic STS confirmed through both pathological and imaging assessments prior to therapy. Additional inclusion criteria were as follows: (1) patients included in the study were diagnosed with advanced or metastatic STS and were not suitable candidates for curative surgery or radiotherapy; (2) at least one measurable lesion according to the Response Evaluation Criteria in Solid Tumors (RECIST) version 1.1; (3) an Eastern Cooperative Oncology Group (ECOG) performance status of 0-2; (4) a left ventricular ejection fraction of 50% or greater, along with adequate bone marrow, liver, and renal function; and (5) a life expectancy of at least 12 weeks.

Exclusion criteria included: (1) individuals with clinically active brain metastases; (2) subjects received prior therapy with anti-angiogenic TKIs; (3) patients with malignancy over the past five years, except controlled squamous or basal cell carcinoma of the skin, or carcinoma in situ of the cervix; (4) patients with active ulcers, major bleeding, or a tendency to bleed.

The study protocol was approved by Institutional Review Boards and independent ethics committees. Additionally, the study was designed and conducted in accordance with Good Clinical Practice and the Declaration of Helsinki. This clinical trial has been registered with ClinicalTrials.gov under the identifier NCT05142631.

**Treatment**

Eligible participants were administered fruquintinib therapy at a dose of 5 mg per day, following a 28-day treatment cycle of 3 weeks on followed by 1 week off until disease progression, patient death, unacceptable toxicity, withdrawal of consent by the patient, or discontinuation by the physician. A protocol-predefined dose reduction in increments of 1 mg was permitted for patients experiencing severe treatment-related toxic effects, and treatment interruptions were allowed to manage fruquintinib-associated adverse events (AEs). Fruquintinib therapy will be discontinued permanently if toxicity does not resolve after a 2-week interruption or failed to meet protocol-defined criteria by two dose reductions.

**Endpoints**

The primary endpoint was PFS, defined as the duration from treatment start to disease progression or patient death due to any cause. The secondary endpoints included ORR (defined as the proportion of patients with complete response (CR) or partial response (PR)), disease control rate (DCR) (defined as percentage of patients who achieved CR, PR, or stable disease (SD)), OS (defined as the time from treatment initiation to death or last follow-up), and safety. The treatment response was evaluated by contrast-enhanced CT and/or MRI after every 8 weeks as per RECIST version 1.1.

Fruquintinib-related AEs were graded according to the National Cancer Institute’s Common Terminology Criteria for Adverse Events (NCI-CTCAE) version 5.0. Follow-up duration was calculated from a participant's entry into the study until the cut-off date or death. At scheduled study visits, duration, severity, and relationship to fruquintinib were documented according to the investigator's clinical assessment.

Additionally, we calculated the baseline NLR, LMR, PLR, SII, and SIRI as exploratory endpoints to investigate their association with PFS and OS in subjects with receiving treatment with fruquintinib.

**Statistical analysis**

PFS and OS were estimated by Kaplan-Meier analysis with 95% confidence intervals (CIs), and log-rank test was applied to compare Kaplan-Meier curves between groups. The Clopper-Pearson method, which is based on the binomial distribution, was used to estimate the ORR and DCR along with their 95% CIs. Cox proportional hazards regression was applied to model the relationship between key variables and patient outcomes. Efficacy and safety were evaluated in all patients who received treatment. Descriptive statistics were used for descriptive data. All analysis were conducted using R software version 4.4.2 and Graphpad 9.0.0, with the database cutoff date set to November 26, 2024.
