# Supplementary material for: Fruquintinib as first‐line or second‐line treatment in unresectable or metastatic soft‐tissue sarcoma: A prospective, single‐arm phase II study
Source: Clin Transl Med. 2025 Apr 15;15(4):e70308. doi: 10.1002/ctm2.70308 (PMC12000221; doi:10.1002/ctm2.70308)
Supplement: Supplementary file 2 — Supporting Information [file CTM2-15-e70308-s002.docx]

**Table S1. Baseline characteristics of patients in the study population**

| Category | STS (n=31) |
| --- | --- |
| Age, median, years | 51 (19-78) |
| Age, years, n (%) |  |
| <60 | 21 (67.7%) |
| ≥60 | 10 (32.3%) |
| Gender, n (%) |  |
| Male | 15 (48.4%) |
| Female | 16 (51.6%) |
| ECOG PS, n (%) |  |
| 0 or 1 | 30 (96.8%) |
| 2 | 1 (3.2%) |
| Histologic type, n (%) |  |
| AS | 6 (19.4%) |
| EHE | 12 (38.7%) |
| HPC | 3 (9.7%) |
| PMHE | 1 (3.2%) |
| SFT | 9 (29.0%) |
| Primary tumor location, n (%) |  |
| Trunk | 6 (20.0%) |
| Extremity | 5 (16.7%) |
| Lung | 6 (20.0%) |
| Brain | 5 (16.7%) |
| Head | 4 (13.3%) |
| Spleen | 3 (10.0%) |
| Breast | 1 (3.3%) |
| Metastases, n (%) |  |
| Yes | 22 (71.0%) |
| No | 9 (29.0%) |
| Number of metastases, n (%) |  |
| 1 | 7 (31.8%) |
| ≥2 | 15 (68.2%) |
| Metastasis site, n (%) |  |
| Lung | 14 (63.6%) |
| Bone | 11 (50.0%) |
| Lymph node | 8 (36.4%) |
| Liver | 6 (27.3%) |
| Pleura | 3 (13.6%) |
| Adrenal | 1 (4.5%) |
| Muscle | 2 (9.1%) |
| Penile | 1 (4.5%) |
| Prior surgery, n (%) |  |
| Yes | 18 (58.1%) |
| No | 13 (41.9%) |
| Prior chemotherapy, n (%) |  |
| Yes | 12 (38.7%) |
| No | 19 (61.3%) |
| Prior radiotherapy, n (%) |  |
| Yes | 4 (12.9%) |
| No | 27 (87.1%) |
| NLR, median (IQR) | 2.6 (1.7-3.5) |
| PLR, median (IQR) | 147.5 (115.4-172.1) |
| LMR, median (IQR) | 3.9 (2.4-4.9) |
| SII, median (IQR) | 555.0 (456.5-809.7) |
| SIRI, median (IQR) | 1.1 (0.9-1.8) |
| Death, n (%) |  |
| No | 19 (61.3%) |
| Yes | 12 (38.7%) |

Abbreviation: AS, angiosarcoma; EHE, hemangioendothelioma; HPC, hemangiopericytoma; IQR, interquartile range; NLR, neutrophil-to-lymphocyte ratio; LMR, platelet-to-lymphocyte ratio; PLR, lymphocyte-to-monocyte ratio; PMHE, pseudomyogenic hemangioendothelioma; SFT, solitary fibrous tumor; SII, systemic immune inflammation index; SIRI, systemic inflammation response index; STS, soft tissue sarcoma.
